# Study on self-management of real-time and individualized support in stroke patients based on resilience: a protocol for a randomized controlled trial

**DOI:** 10.1186/s13063-023-07475-x

**Published:** 2023-08-03

**Authors:** N. Jiang, Y. Xv, X. Sun, L. Feng, Y. B. Wang, X. L. Jiang

**Affiliations:** 1 West China School of Nursing/West China Hospital, Sichuan University, Chengdu, 610041 Sichuan China; 2 Department of Surgical Anesthesiology, Tai’an City Central Hospital, Tai’an, China; 3 Department of Neurology, Tianjin Third Central Hospital, Tianjin, China; 4 Department of Neurology/West China School of Nursing, West China Hospital, Sichuan University, Chengdu, China; 5 Tai’an Tax Bureau, State Administration of Taxation, Tai’an, China

**Keywords:** Self-management, Real-time support, Wearable devices, Stroke patients

## Abstract

**Background:**

The transitional period from hospital to home is vital for stroke patients, but it poses serious challenges. Good self-management ability can optimize disease outcomes. However, stroke patients in China have a low level of self-management ability during the transitional period, and a lack of effective support may be the reason. With the rapid development of technology, using wearable monitors to achieve real-time and individualized support may be the key to solving this problem. This study uses a randomized controlled trial design to assess the efficacy of using wearable technology to realize real-time and individualized self-management support in stroke patients’ self-management behavior during the transitional period following discharge from hospital.

**Methods:**

This parallel-group randomized controlled trial will be conducted in two hospitals and patients’ homes. A total of 183 adult stroke patients will be enrolled in the study and randomly assigned to three groups in a 1:1:1 ratio. The smartwatch intervention group (*n* = 61) will receive Real-time and Individualized Self-management Support (RISS) program + routine care, the wristband group (*n* = 61) will wear a fitness tracker (self-monitoring) + routine care, and the control group (*n* = 61) will receive routine stroke care. The intervention will last for 6 months. The primary outcomes are neurological function status, self-management behavior, quality of life, biochemical indicators, recurrence rate, and unplanned readmission rate. Secondary outcomes are resilience, patient activation, psychological status, and caregiver assessments. The analysis is intention-to-treat. The intervention effect will be evaluated at baseline (T0), 2 months after discharge (T1), 3 months after discharge (T2), and 6 months after discharge (T3).

**Discussion:**

The cloud platform designed in this study not only has the function of real-time recording but also can push timely solutions when patients have abnormal conditions, as well as early warnings or alarms. This study could also potentially help patients develop good self-management habits through resilience theory, wearable devices, and individualized problem–solution library of self-management which can lay the foundation for long-term maintenance and continuous improvement of good self-management behavior in the future.

**Trial registration:**

The ethics approval has been granted by the Ethics Committee of West China Hospital, Sichuan University (2022–941). All patients will be informed of the study details and sign a written informed consent form before enrollment. The research results will be reported in conferences and peer-reviewed publications. The trial registration number is ChiCTR2300070384. Registered on 11 April 2023.

**Supplementary Information:**

The online version contains supplementary material available at 10.1186/s13063-023-07475-x.

## Introduction

Stroke has become one of the major diseases endangering the health of individuals around the world, with the characteristics of high incidence, high disability rate, high mortality, and high recurrence rate. It is the third most deadly disease in Western countries [1], and it is also the leading cause of death and disability in Chinese adults [2]. With the rapidly aging population in China, the burden of stroke disease presents an explosive growth trend [2, 3].

The transition period from hospital to home is a very important part of disease care for stroke patients after discharge, but it faces severe challenges such as decreased self-care ability, lack of knowledge, poor treatment compliance, and lack of adequate follow-up support and service continuity, which leads to the increased risk of early readmission after discharge and endangers the safety of patients [4–6]. There are two major health tasks in the transition period of stroke patients. The first is functional rehabilitation, including rehabilitation exercise, drug therapy, diet management, and emotional management. The second is to prevent recurrence [7, 8]. These tasks are closely related to patients’ self-management behaviors. The better patients control their self-management behavior, the better their outcome indicators in terms of neurological recovery, ability to perform activities of daily living, and social ability [9]. However, studies have shown that the self-management ability of Chinese stroke patients remains at a lower-moderate level [10, 11]. Lack of effective support is the main reason [12]. With the rapid development of wearable technology, real-time and accurate self-management support using wearable technology may be the key to solving this problem [13, 14]. However, we found that the functions of wearable devices involved in stroke research were mainly intelligent rehabilitation training devices, and they were mostly used for rehabilitation treatment, mainly focusing on the recovery training of limb function [15], sensory stimulation [16], swallowing function [17], and support for cognitive impairment [18]. There were relatively few studies on the monitoring of physical indicators (such as heart rate, blood pressure, electrocardiogram, sleep) and self-management behaviors (such as exercise, medication behavior, diet management) of stroke patients. As a disease with a high disability rate and high recurrence rate, how to combine wearable devices with disease monitoring and health behavior promotion is worthy of further exploration by researchers. There are various types of wearable devices, and with the development of sensors, integrated chips, and intelligent interaction technology, the measurement speed and accuracy of wearable devices are being perfected. Common wearable devices used for stroke include smart insoles [19], leg assist devices [15], rehabilitation sportswear [20], arm bands [21], smart gloves [22], sleeve covers [23], and smart bracelets [24]. Most wearable devices used for disease monitoring are smart bracelets and watches, which are more easily accepted by patients because of their portability and affordability. In sum, most studies take the functional recovery of a certain body part as the outcome index, and few studies focus on the overall well-being of patients and how wearable devices can help them achieve accurate physical and mental care.

Transition management refers to a series of planned behaviors taken to ensure the coordination and continuity of healthcare services when patients are transferred between different healthcare institutions or different healthcare departments in the same institution [25]. In terms of research on self-management interventions for stroke patients during the transition period, most studies have used rehabilitation training, health education, and disease monitoring as the main intervention measures [26, 27]. There is a lack of research on the family environment and community resource utilization. The intervention time ranged from 4 weeks to 9 months, and the intervention intensity gradually decreased over time [28–31]. Intervention strategies included psychological intervention, behavioral intervention, rehabilitation intervention, and treatment compliance. The greater the number of intervention strategies, the greater the impact on patient outcomes [32]. In terms of interventions, multidisciplinary joint intervention is the main method. Compared with a single professional intervention team, a multidisciplinary intervention team can better meet the diverse healthcare needs of stroke patients [33]. In summary, the implementation of the transitional self-management intervention project has achieved certain effects in improving the self-efficacy, quality of life, and treatment compliance and reducing the recurrence rate of patients. However, shortcomings remain: first, the patients’ condition cannot be monitored in real time and accurately after discharge; second, as a disease with a high recurrence rate, it is not possible to effectively warn patients about all possible abnormal conditions. Third, the duration of interventions studied was different, and the timeliness of transition management was ignored. Fourth, universal intervention strategies were adopted for all patients, and individualized intervention measures were not implemented according to the dynamic changes in patients.

Social-ecological resilience (SER) is the ability of individuals to adapt well in the face of adversity and to obtain psychological, social, cultural, and material resources to maintain and restore health. SER includes four levels: individuals, external relationships, community structure, and spirit/culture [34]. For stroke patients, the main difficulties they face are the inconvenience of physical activity caused by neurological deficits, social limitations, increased family dependence, and bad emotions caused by poor recovery, which involve patients’ physiological, psychological, family, and social aspects [35]. Studies have shown that good resilience can effectively promote the self-management ability of stroke patients [36]. The solution-focused approach (SFA) is an important embodiment of resilience theory. It takes finding solutions to problems as the core, focuses on individual advantages, and stimulates the potential ability of patients to solve problems by excavating their own strengths [37]. For chronic disease nursing, the SFA emphasizes that nurses should shift from disease care to helping patients improve adaptability and resilience, stimulating patients’ active participation by exploring their potential and external resources, and ultimately improving patients’ self-management ability [38]. Studies have applied SFA to mental disorders, diabetes, obesity, craniocerebral injury, and other diseases [39–41] and have achieved good results. However, studies on the combination of SAF and wearable technology in the transitional management of stroke patients are rare.

In summary, on the one hand, stroke patients in the transition stage are seriously ill, prone to recurrence, and have poor self-management ability, which requires close monitoring. However, the effect of measures on improving patients’ self-management ability is not ideal, and insufficient support is the bottleneck. On the other hand, wearable technology has been gradually applied in medical research, which has promoted the diagnosis, treatment, and continuous monitoring of diseases. However, there are few reports on how to use wearable technology to provide real-time support for stroke patients who are at home during the transition period and need to pay close attention to changes in their condition. So, how to combine wearable technology in the transition period with real-time support for patients’ self-management is research worthy of further exploration.

## Methods

### Study design

**Fig. 1:**
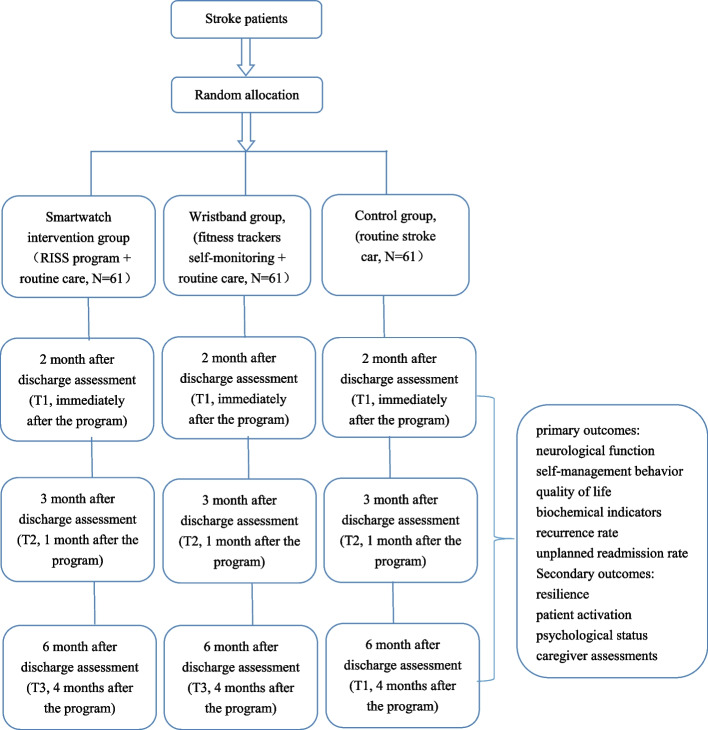
The summary of the study design

**Fig. 2:**
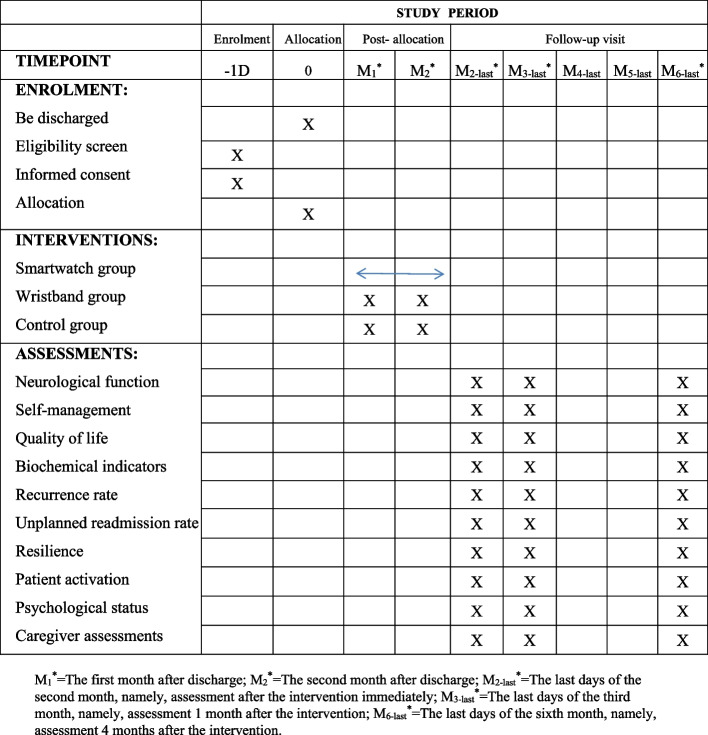
Schedule of enrollment, interventions, and assessments

This study is a parallel-group randomized controlled trial with a 1:1:1 randomization scheme that will be conducted in West China Hospital of Sichuan University and Chengdu Second People's Hospital from April 2023 to April 2024. In this study, the Real-time and Individualized Self-management Support (RISS) program will be developed for stroke survivors guided by resilience theory. Patients will wear smartwatches (MLY B10, Huawei wrist electrocardio-blood pressure record) that can collect real-time data such as exercise, sleep, blood pressure, heart rate, and blood oxygen to prompt self-management support for transitional stroke patients. Our hypothesis is that compared with the wristband group and control group, stroke patients who receive the RISS program will have a lower incidence of recurrence rate and an improved quality of life. This study could provide new ideas for increasing patients’ self-management ability, improving quality of life, and optimizing disease outcomes. This trial protocol uses the Standard Protocol Items: Recommendations for Interventional Trials (SPIRIT) reporting guidelines (see Additional file 2). The research method conforms to the joint standard of multiarm test reports. A summary of the study design is shown in Fig. 1. Schedule of enrollment, interventions, and assessments is shown in Fig. 2.

### Participants

Patients with stroke who are about to be discharged from departments of neurology in the West China Hospital of Sichuan University and Chengdu Second People's Hospital, will be recruited and followed up until week 24 after randomization. Trained investigators and research assistants will approach and inform patients about the study protocol, the randomization process, and the details of the intervention. Participants will sign an informed consent form prior to participation in the study. The inclusion criteria are as follows: (1) meeting the diagnostic criteria of ischemic stroke, confirmed by brain CT or magnetic resonance imaging; (2) stable condition; (3) muscle strength grade II or above, Barthel Index > 40; (4) age ≥ 18 years old; (5) clear consciousness and ability to communicate with researchers through writing/language; (6) residence in Chengdu; (7) discharge home rather than to other care institutions; (8) ability to use a mobile phone and WeChat; and (9) voluntary participation in the study.

The exclusion criteria are as follows: (1) previous history of mental illness or existing mental disorder and (2) serious concomitant diseases, such as severe hepatic and renal insufficiency, severe arrhythmia, frequent angina pectoris, heart failure, and myocardial infarction.

### Recruitment

The recruitment sites for this research are the departments of neurology, West China Hospital of Sichuan University and department of neurology, Chengdu Second People's Hospital. They are all in Chengdu, Sichuan province, China. Each day from 15:00 to 17:00, the research assistants will go to the nurses’ station to collect the basic information of patients who are going to be discharged the next day and screen patients according to the inclusion and exclusion criteria. Then, the research assistants will invite the patients to the nurse station for communication and explain the purpose, significance, specific implementation process, and precautions of the project and invite patients to participate in this study. After obtaining the patient’s consent, the research assistant will record the patient’s basic information (i.e., name, sex, age, disease condition) and contact information and invite patients to join the study’s WeChat group. For the purpose of intention-to-treat analysis, if a participant drops out of the study, the research assistant will also record their basic information and the reasons for withdrawal for comparison with the enrolled participants.

### Randomization and blinding

Patients who are scheduled to be discharged from the hospital each day will be randomly assigned to the smartwatch intervention group, the wristband group, and the control group at a ratio of 1:1:1. The randomization process is as follows: According to the predetermined number 1 for the smartwatch intervention group, number 2 for the wristband group, and number 3 for the control group, a slip of paper with numbers 1, 2, and 3 will be placed into a sealed, opaque envelope by a research assistant who is unaware of the design of the study. The number selected will determine the group to which the patient will be assigned until 61 subjects are in each group. Because participants in different groups may be in the same hospital room, all participants in the smartwatch intervention group and the wristband group will sign a confidentiality agreement stating that the study will not be disclosed to participants in the control group. Research assistants will generate the allocation sequence and enroll participants, and the researcher will assign interventions.

Because of the nature of the intervention, intervention practitioners cannot be blinded, but patients, data collectors, and statisticians will be blinded to the intervention allocation. In addition, the outcomes of this study will be objectively measured and not influenced by the unblinded intervention. Unblinding will be done after the study.

### Sample size

According to Polit and Beck [42], when using ANOVA to compare the means of multiple groups, the simplest method to calculate the sample size is to estimate the ratio of the sum of squared deviations between the groups and the sum of squared deviations of the total (eta-squared, *η*2). However, when there is no experimental result of the same kind of study to base *η*2, the researcher can predict whether the study effect is large, medium, or small. According to the convention, ANOVA is used to compare the means of the smartwatch group, wristband group, and control group. The *η*2 values of large, medium, and small effects are 0.14, 0.06, and 0.01, respectively. The corresponding sample sizes of the three groups are 22, 53, and 319, and the sample size of each group is 22, 53, and 319 when *α* = 0.05 and *β* = 0.02, respectively. Because there were no suitable previous research results as the basis for calculating sample size by formula, the sample size is determined by the estimation method. Taking *α* = 0.05 and *β* = 0.02, the effect is estimated to be medium, and the number of samples in each group is 53. Considering the loss of samples and other reasons to increase the sample content by 15%, we finally included 61 cases in each group for a total of 183 subjects.

### Study interventions

Subjects in the smartwatch intervention group will receive the RISS program + routine care, subjects in the wristband group will receive exercise wristbands (Huawei Bracelet 6 FRA-B29) + routine care, and subjects in the control group will receive routine stroke care. Specific interventions for the three groups are as follows:

- 1. Smartwatch intervention group:
  1. Build a real-time and individualized support system for stroke patients with a cloud platform as the core.

**Fig. 3:**
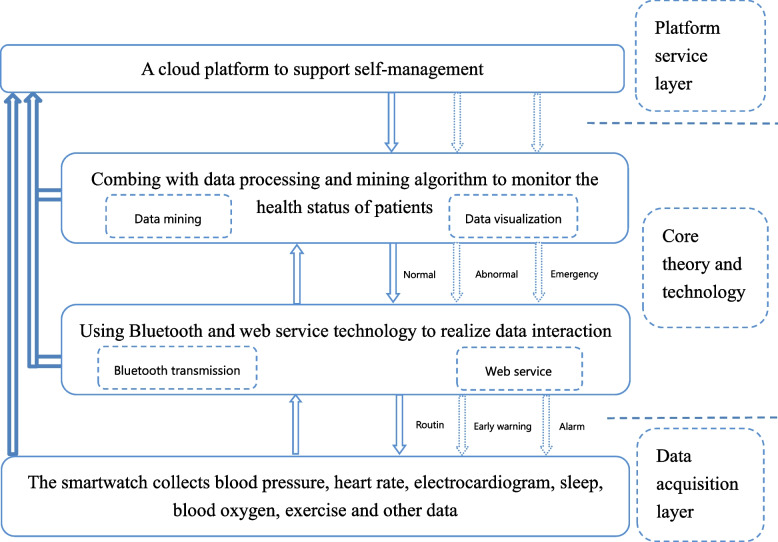
The real-time self-management support cloud platform system for stroke patients

The system is composed of three parts: a data acquisition layer, a data transmission layer, and a platform service layer. (1) Data acquisition layer—a medical-grade smartwatch will be used, which has the functions of measuring blood pressure (balloon type), heart rate, and electrocardiogram patterns and screening for arteriosclerosis risk and sleep disturbances including sleep apnea, blood oxygen saturation, body temperature, Global Positioning System (GPS), and exercise state. The device has been registered as a class II medical device by the National Medical Products Administration and has good stability and accuracy. (2) Data transmission layer: the smartwatch will be connected to the patient’s personal phone, and the mobile app will be downloaded. The patient’s health data collected by the smartwatch will be transmitted to researchers via Bluetooth in real time. The app can realize family spatial data sharing so that family members can check the patient’s blood pressure and heart rate at any time, and the data in the mobile app can be transmitted to the platform service layer through web service technology. (3) Platform service layer—health data such as blood pressure, heart rate, and electrocardiogram patterns collected in real time will be uploaded to the database of the platform server and processed by the *k*-means clustering method; the calculations will be transmitted in real time to assist the medical staff of the background research team in supporting patients’ self-management. A “common problem–solution database” for stroke patients is set up in the platform. When the data monitored by the smartwatch is normal, researchers will intervene according to the problems that may occur in the routine stages of the disease and any individual problems a patient may have. When the data exceeds the warning value, the warning signal will trigger the abnormal situation processing scheme library, and the platform will push the corresponding solutions to the mobile phone of the patient and his or her family and send a warning to the medical staff, who will take measures to help the patient recover to his or her normal state as soon as possible. When the patient’s emergency reaches the alarm limit, the platform will push the first aid plan to the patient and his or her family and remind them to dial 120 and notify the medical staff about the emergency (Fig. 3).

- (2) Implementation of intervention
  - ① Intervention activities before discharge

Time: the day before discharge

Location: hospital

Objective: to help subjects analyze the status of self-management, identify individual problems, formulate self-management goals together with subjects, and understand the use of smartwatches and precautions.

Content: (a) The subjects are given home self-management health guidance manuals, which include medication compliance, rehabilitation training precautions, psychological adjustment, symptom self-detection, diet adjustment, life, work and rest, regular medication, and regular follow-ups, and they are also given discharge guidance. The main content teaches compliance with the doctor’s advice after discharge, health lifestyle education, and self-management status analysis. At the same time, the subjects will keep home diaries. Evaluating subjects before discharge to determine their self-management-related problems in the four domains of environmental, psychosocial, physiological, and health-related behaviors; and to formulate health goals after discharge with the subjects/caregivers. (b) Demonstrate the use of smartwatches, explain the precautions in the use of smartwatches, and teach the subjects to use smartwatches skillfully.

② Intervention activities after discharge

Time: the intervention will last for 6 months after discharge, and the intervention is conducted twice a week in the first 2 weeks at home, once online, and once offline. The intervention will be conducted once a week from the 3rd to the 8th week, online and offline are combined, and patients are followed up at the end of 2 months, 3 months, and 6 months after discharge.

Location: home

Objective: to provide targeted and individualized interventions for subjects’ problems and reduce complications.

Content:

a Developing a common problem–solution library for stroke patients’ self-management.

Prior to the intervention, a cross-sectional survey and semi-structured interview will be conducted among stroke patients in transition to form a common problem set on self-management. Based on the set, a common problem–solution library (CPS) for stroke patients will be established according to various stroke guidelines. We will invite 8–12 experts to evaluate the initial version of the CPS and plan to finalize the revised version through 2–3 rounds of expert consultation.

b Developing an individualized problem–solution library for stroke patients’ self-management.

During the intervention period, for patients in the intervention group, objective data (blood pressure, heart rate, electrocardiogram, and other objective data collected by smartwatch) and subjective data (semi-structured interviews with patients, caregivers, medical staff, community workers, and other relevant people who may affect patients’ self-management behavior) will be analyzed before each intervention to identify individual problems of each patient. Then, according to the CPS and combined with the patient’s own characteristics, the medical staff will carry out individualized interventions on the problems of each patient according to the five steps of the RISS program, namely, describing the problem, establishing feasibility goals, exploring patients’ advantages and providing individualized interventions, giving feedback, and evaluating progress (Supplementary file 1). After completion of each intervention, the medical staff will record the problems, measures taken, and effect analysis of each patient in detail in the form of “disease management log” on the administrator end of the cloud platform. After 8 weeks of about 10 consecutive assessments and interventions, the problems existing in the transitional period of patients might be saturated, and the solution library for these problems is also established, which is the individualized problem–solution library (IPS) for self-management.

After the completion of the last intervention, we will send the CPS and the IPS to each patient in the form of a guidance manual and assist patients to make a self-management supervision sheet, so that patients can identify their problems in self-management and individualized solutions. It can point out the direction for the long-term maintenance and continuous improvement of good self-management behavior of patients in the future.

- 2. Wristband group

The subjects in the bracelet group will wear exercise wristbands that can record heart rate, sleep, blood oxygen, and exercise in real time, and the subjects will conduct self-testing at home. At the same time, the subjects will be given routine discharge care, that is, telephone follow-up twice in the first week and the fourth week after discharge. The content will include the implementation of the discharge doctor’s advice, inquiry of physical function recovery, health advice for common problems, and rehabilitation guidance.

- 3. Control group

Subjects in the control group will receive routine discharge care, that is, telephone follow-up twice in the first week and the fourth week after discharge, which will include the implementation of the discharge doctor’s advice, inquiries about physical function recovery, health advice for common problems, and rehabilitation guidance.

### Evaluation methods

An assessor-blind method is used; that is, questionnaire collectors are research assistants who are trained in the use of measurement tools but are blinded to the grouping of study subjects. The intervention effects of the three groups of patients will be measured at baseline and 2 months, 3 months, and 6 months after discharge. The evaluation content includes questionnaire surveys and biochemical index measurements. Baseline data (during hospitalization) collection: Patients will complete the assessment at the nurses’ station. Follow-up data (after discharge from the hospital) will be collected as follows: The research assistant will visit the patient at home for data collection. Patients will complete the assessment independently, but those with reading difficulties, visual impairment, or handwriting difficulties will be instructed to complete the questionnaire as each item is read to them in neutral, noncryptic language. After the evaluation, the researchers will check whether the evaluation is complete. If there are missing items, patients will complete the items on the spot and return the evaluation for verification again. Any doubts about the content will be verified with the patient on the spot. Upon completion of data collection at each follow-up time point, each study patient will receive a gift for participating in the study. Basic demographic data will be collected at baseline, including age, sex, ethnicity, education level, marital status, job status, family status, and medical condition.

- 1. Primary outcome (for patients)

The primary outcomes are neurological function status, self-management behavior, quality of life, biochemical indicators, recurrence rate, and unplanned readmission rate.

1. Neurological status

The National Institutes of Health Stroke Scale (NIHSS) is the most common stroke evaluation index in the world. It covers the anterior and posterior circulation of the brain, including consciousness, movement, sensation, speech, and visual field, with a total of 15 items. NIHSS scores range from 0 to 42, with higher scores indicating more severe neurological impairment [43]. Cai Yefeng et al. introduced the NIHSS in China to conduct a multicenter and large sample test, and the results showed that the Chinese version of the NIHSS had good reliability, validity, and sensitivity [44].

The Modified Barthel Index (MBI) will be used to assess the patient’s ability to perform ADLs. The scale contains 10 items: eating, grooming (washing face, brushing hair, brushing teeth, shaving), bathing, dressing (unfastening buttons, zipping zippers, putting on shoes and socks), bowel and urine control, toileting, bed-chair transfer, moving on the ground, and walking up and down stairs [45]. Each item is rated on a 1–5 scale as completely dependent, needing a lot of help, needing moderate help, needing little help, and being completely independent. Normal is 100 points, with a score of 91–99 indicating mild dependence; 61–90, moderate dysfunction; 21–60, severe dysfunction; and 0–20 points, completely dependent. The Chinese version has good reliability and validity [46].

- (2) Self-management behavior: The Stroke Self-management Behavior Rating Scale (SSBR) will be used to measure the level of self-management behavior of stroke patients. The scale covers 7 dimensions: disease management, medication management, diet management, daily life management, emotional management, social function and interpersonal management, and rehabilitation exercise management, with a total of 51 items. Each item is scored from 1 to 5 points, and the total score of the scale ranges from 51 to 255. The higher the score, the better the self-management behavior of the patients. The Cronbach’s *α* coefficient is 0.847, and the construct validity is 0.761. The scale has been widely used in China and has good reliability and validity [47].
- (3) Quality of life: The 12-item Medical Outcomes Study Short Form Health Survey version 2.0 (SF-12 v2) developed by Boston Health Research Institute will be used to measure the quality of life of patients. It has eight dimensions: general health (GH), physical functioning (PF), role physical (RP), bodily pain (BP), vitality (VT), social functioning (SF), role emotional (RE), and mental health (MH), with a total of 12 items. The total score of the GH, PF, RP, and BP dimensions is the physical component summary (PCS). The total score of the SF, RE, MH, and VT dimensions is the mental component summary (MCS), and the standardized score of each part ranges from 0 to 100. The higher the score, the better the quality of life of the patient. The total Cronbach’s *α* coefficient of the Chinese version of the questionnaire is 0.84, and Cronbach’s *α* coefficient of each dimension is greater than 0.70 [48].
- (4) Recurrence rate: In China, the recurrence rates of stroke patients at 3 months, 6 months, and 1 year are 12.9%, 16.0%, and 17.7%, respectively [49]. The mortality and disability rate after the recurrence of stroke will be higher, which creates a more serious burden for patients and their families. Therefore, the recurrence rate of stroke after discharge will be used as one of the evaluation indicators in this study.
- (5) Biochemical indicators: Blood glucose and blood pressure are important risk factors for stroke recurrence [50, 51]. In this study, the blood glucose and blood pressure of patients will be measured by a stable glucose meter (Sinocare Anzhun) and an Omron sphygmomanometer (Sinocare BA-802).
- (6) Unplanned readmissions: Unplanned readmissions for stroke patients are defined as unplanned readmissions to the department of neurology of a hospital for similar conditions within 30 days or less from the date of last discharge to the date of readmission. It is calculated using the following formula [52]: unplanned readmission rate = number of unplanned readmissions/total number of discharges in the group. The Health Commission of China considers unplanned readmission of stroke patients as one of the sensitive indicators of hospital return and an important indicator to evaluate the quality of hospital care [53].

- 2. Secondary outcomes (for the patient)

The secondary outcomes include resilience, patient activation, psychological status, and main caregiver evaluation indicators, including caregiver burden, family resilience, and social resource utilization.

- (1) Resilience: The 10-item Connor and Davidson Resilience Scale (CD-RISC 10) contains a total of 10 items, which are divided into two dimensions: strength and resilience. The total score of the scale is the sum of the scores of each item. The higher score indicates a higher level of resilience [54]. Cronbach’s *α* of the Chinese version of the scale is 0. 922 [55].
- (2) Patient activation: The patient activation scale consists of 13 items, and the scores range from 0 to 100 points using Lister’s 5-point scale. The higher the score, the higher the patient activation level [56]. Cronbach’s *α* coefficient of the Chinese version of the scale is 0.88 [57].
- (3) Social Support: The Multidimensional Scale of Perceived Social Support (MSPSS) consists of three dimensions: significant other, family, and friends, with a total of 12 items. The scores range from 12 to 84, with higher scores indicating more social support [58]. Cronbach’s *α* coefficient of the Chinese version is 0.896 [59].
- (4) Depression: The Patient Health Questionnaire-9 (PHQ-9) contains 9 entries, each with a score of 0–3. The questionnaire is assessed according to the sum of items, and the higher the score, the higher the degree of depression [60]. This questionnaire has been widely used in the world, and the Chinese version of Cronbach’s *α* coefficient is 0.743 [61].

- 3. Secondary outcomes (for informal caregivers)
  - (1) Caregiver burden: The Caregiver Burden Scale consists of two dimensions, individual burden and responsibility burden, with a total of 22 items. The higher the score, the heavier the burden of caregivers [62]. Cronbach’s alpha coefficient of the Chinese version of the scale is 0.96 [63].
  - (2) Family resilience: The Family Resilience Scale is used to assess resilience within the family. The scale includes three dimensions of commitment, challenge, and control, with a total of 20 items. Lister’s 4-point scale will be used, and higher scores indicate better family resilience [64]. Cronbach’s *α* coefficient of the Chinese version of the scale is 0.803 [65].
  - (3) Social resource utilization: The chronic illness resource scale was developed by Glasgow et al. [66], and Yao Xiaoyue et al. [67] revised it. The scale is used to measure the support of patients from seven resources, such as personal, health management team, family/friends, community/neighborhood, media/policy, and workplace, for common self-management behaviors of patients, such as diet, exercise, and medication management behaviors. Based on this scale, we will conduct interviews on the availability of different resource levels.

- 4. Substudy

As a substudy of this trial, we will use cost-effectiveness analysis to evaluate the cost and effectiveness of transition management of stroke patients based on wearable devices. Health economics indexes are evaluated by cost, cost-effectiveness analysis, and sensitivity analysis. The cost-effectiveness of the smartwatch group, control group, and bracelet group are compared with each other to provide a reference for realizing the optimal allocation of medical resources.

### Data management and statistical analysis

The data collector guided the patients to complete the questionnaire and collected the objective data. After patients complete the questionnaire, it will be checked on the spot. If there are any unclear or missing items, the questionnaire will be corrected with the research subject on the spot. In the data entry stage, a unique identification number will be assigned to each patient, and two research assistants will use the Epidata V.3.1 software to enter and double-check the data. Any unclear or missing information will be cross-checked with the original documents and medical records. Data entry personnel will be blinded. The data in the paper questionnaire will be entered into the computer on the same day. After the data entry is completed, the paper questionnaire will be sealed in the office data storage cabinet of West China College of Nursing, and the data will be copied to the encrypted mobile hard disk and desktop computer at the same time to prevent data loss. The coordinating center is affiliated to the West China Nursing School of Sichuan University. It consists of a department chair, two staff members, a postdoc, and four graduate students. They coordinate with hospital departments and communities to ensure patient recruitment and home-based interventions. The trial steering committee is the clinical trial review team of the Ethics Committee of West China Hospital of Sichuan University (including clinicians, nurses, and full-time scientific researchers). The first review by the review team takes place prior to patient recruitment to ensure that the methods used to recruit patients meet the statistical and feasibility requirements. After that, the review team meets every 6 months to monitor until the trial is completed. At the end of the trial, a conclusive review will be conducted. During this time, they will assess the integrity of the trial process to ensure that the parties concerned are fully fulfilling their tasks and responsibilities. The data monitoring committee is composed of 3–5 staff members of the Ethics Committee of West China Hospital of Sichuan University, who will monitor the study data every 1 year. The coordinating center, trial steering committee, and data monitoring committee are independent of the sponsor and have no conflicts of interest.

The SPSS 22.0 statistical analysis software will be used to analyze the data, and *P* < 0.05 (two-sided test) will be considered statistically significant. Count data are described by frequency/constituent ratio, and measurement data are described by mean/standard deviation according to the results of the normality test. Pearson’s chi-square test will be used to compare the count data between groups. One-way analysis of variance will be used to compare the measurement data between the three groups, and the Bonferroni method will be used for further comparison between the two groups. Repeated measures analysis of variance will be used to compare the longitudinal changes in indicators with a normal distribution. If the covariance matrix of the observed values at each time point is symmetric or spherical (Mauchly’s test, *P* ≥ 0.05), no correction will be needed. If the covariance matrix of the observed values at each time point is *P* < 0.05 (Mauchly’s test), the Greenhouse‒Geisser correction test will be used.

### Ethics and dissemination

The study proposal is approved by the Ethics Committee of West China Hospital, Sichuan University (Approval No. 2022–941). Patients will be advised in detail about the purpose, process, possible benefits, and risks of the study. Participation in the study is completely voluntary, and patients can refuse to participate in the study or withdraw from the study at any stage without reason. Their medical treatment and rights and interests will not be affected. All questionnaires, protocols, and results used in the study will be stored in a secure place or with a secure password and will be destroyed within 5 years after the end of the study. No personal patient information will be disclosed in any public report. Every effort will be made to protect the privacy and personal information of the participants’ medical data. This study is focused on health education, and adverse events may include unexpected symptoms or illnesses; whether or not they are related to this study, they will be recorded and reported. Any serious adverse events will be reported immediately to the ethics committee. In case of research-related injury, we will provide free treatment and compensation according to the law. At the end of the study, the follow-up will be terminated when the patients’ blood pressure fluctuated smoothly, and there are no obvious abnormalities in heart rate, electrocardiogram, and sleep for more than 1 month. This study does not involve additional treatment or tests and does not interfere with the patients’ normal treatment. However, when the patient becomes sicker, the study leader will decide to terminate the patient’s participation in order to ensure that the patient receives timely treatment. The findings will be published in a peer-reviewed research journal and presented at conferences in accordance with CONSORT recommendations.

### Improving adherence to interventions, modifications, and post-trial care

To improve adherence to interventions in each intervention, we repeatedly emphasized the importance of wearing smartwatches to monitor physical signs for patients’ recovery. And the study required patients to upload self-management logs to the cloud platform every other day. The system will have a built-in reminder module to remind patients through WeChat and mobile phone messages. As a reward, patients who upload their reflection logs on time each week will receive a small gift. This study focuses on health education. The medical treatment or rehabilitation methods of patients in the home stage are based on the doctor’s advice, and the study does not involve additional or alternative medical treatment and rehabilitation methods. If the patient has a need during follow-up, the investigator will communicate with the patient’s doctor to provide the patient with a solution to the problem as an alternative treatment or rehabilitation method. After the intervention, the smartwatch will be taken back, but the WeChat group will not be dissolved. Patients can contact the medical staff in the WeChat group for answers if they have any questions.

## Discussion

Most stroke survivors are left with hemiplegia, aphasia, mouth and eye deviations, dysphagia, cognitive impairment, and other functional abnormalities, which require long-term or even lifelong rehabilitation care, which creates a serious burden for patients, families, and society. Studies have shown that the average length of hospital stay for stroke patients is approximately 10 days [68–70]. It is of great significance to obtain continuous, real-time, and precise attention after discharge to promote functional recovery and reduce disability and recurrence rates. However, due to the limitations of medical resources, facilities, equipment, and technology, most real-time and precise management of stroke is limited to patients in the hospital during the acute onset of stroke, and the attention to patients after discharge is often reduced, resulting in a high incidence of adverse outcomes and readmission rates [71]. Therefore, achieving a real-time and precise care model of stroke patients’ self-management during the transition period at a relatively low cost is helpful in improving the overall health service level, which has important clinical significance, academic value, and economic benefits.

This proposed study will evaluate the effectiveness of a resilience improvement intervention program with the help of a wearable device specifically designed for transitional stroke patients. It is an intervention program delivered by trained health professionals in combination with an engineering group. It also provides guidance on self-management behavior for stroke survivors. Due to the lack of previous research on transitional self-management interventions based on wearable devices, under the framework of social ecological theory and resilience theory, we will use SFA to construct a real-time and precise care plan for transitional self-management of stroke patients based on resilience improvement and provide interventions for patients at the individual, relationship, community structure, and culture levels. This model will provide uninterrupted medical services and real-time monitoring for patients. Digital health interventions for patients with wearable devices, on the other hand, require the patient to wear them for a long period of time but may suffer from cost–benefit imbalances and patient uncooperation. This study hopes that by wearing wearable devices for a short period of time, patients can identify problems existing in their own management and individual solutions, so as to form good habits of self-monitoring and self-management. Even if patients are no longer equipped with wearable devices after the intervention, the individualized problem-solving libraries established by the study can also provide directions for patients to maintain high-quality self-management behaviors in the long run.

This study has some limitations. First, the study population is focused on patients with ischemic stroke, and the findings may not be generalizable to patients with hemorrhagic stroke. Second, the main purpose of this study is to improve the self-management ability of patients, that is, patients are required to have a certain degree of self-care ability, so the inclusion criteria required that the BI index be greater than 40, which may have limited the participation of some severely ill patients, and these patients also need our attention. Based on the above limitations, we will extend transition management based on wearable devices to hemorrhagic stroke and critically ill patients in future research. In addition to these limitations, the data collected by the wearable devices used in the study may be inaccurate due to the device or the improper way that patients wore the device. To mitigate these risks, research assistants will repeatedly teach patients and informal caregivers about how to wear and use the device during enrollment, emphasizing the precautions, and allowing patients to practice many times until they become proficient. The wearable device used in this project is a medical-grade smartwatch that can measure blood pressure, heart rate, electrocardiogram, sleep, blood oxygen, exercise status, and other functions. It has been registered as a class II medical device by the China Food and Drug Administration and has good stability and accuracy. At present, research on the “real-time support” care model of stroke patients’ self-management could provide new ideas for breaking through the bottleneck of stroke patients’ insufficient self-management ability. The findings of this work will (1) provide evidence-based information on innovative interventions for patients with stroke during the transition period; (2) offer real-time and precise intervention for patients through wearable technology, stepping out of the original thinking of a single intervention plan, giving patients individualized intervention models based on dynamic patient data, and providing empirical evidence for the application of wearable technology in stroke patients; and (3) stimulate patients’ positive mind set and improve their adaptability in the face of adversity based on resilience theory.

## Trial status

Recruitment had not started when the protocol was submitted. The protocol version is V1, 17 April 2023. The recruitment will begin on May 10, 2023, and will be completed at about April 10, 2024.

### Supplementary Information

**Additional file 1.**

**Additional file 2.**

## Data Availability

Any data required to support the protocol can be supplied on request. The datasets analyzed during the current study and statistical code are available from the corresponding author upon reasonable request, as is the full protocol. Any important protocol modifications will be communicated with investigators, trial participants, trial registries, journals, and regulators.

## Abbreviations

RISS: Real-time and Individualized Self-management Support
SER: Social-ecological resilience
SFA: Solution-focused approach
GPS: Global Positioning System
CPS: Common problem–solution library
IPS: Individualized problem–solution library
NIHSS: National Institutes of Health Stroke Scale
MBI: Modified Barthel Index
SSBR: Stroke Self-management Behavior Rating Scale
SF-12 v2: 12-Item Medical Outcomes Study Short Form Health Survey Version 2.0
PCS: Physical component summary
MCS: Mental component summary
CD-RISC 10: 10-Item Connor and Davidson Resilience Scale
MSPSS: Multidimensional Scale of Perceived Social Support
PHQ-9: Patient Health Questionnaire-9
