# Supplementary material for: Study on self-management of real-time and individualized support in stroke patients based on resilience: a protocol for a randomized controlled trial
Source: Trials. 2023 Aug 3;24:493. doi: 10.1186/s13063-023-07475-x (PMC10401848; doi:10.1186/s13063-023-07475-x)
Supplement: Supplementary file 1 — Additional file 1. [file 13063_2023_7475_MOESM1_ESM.docx]

Intervention methods: According to the five steps of the SFA (describing the problem, establishing feasibility goals, exploring patients’ advantages and providing individualized interventions, giving feedback, and evaluating progress), the patients are intervened by combining quantitative and qualitative research. The intervention process is as follows. Describing the problem: By reviewing and summarizing the data collected by smartwatches + patient self-management diaries + interviews with patients and main caregivers, the self-management problems of subjects at this stage are clarified. b. Establishing feasibility goals: According to the problems of the patients this week, the miracle question method is used to explore the ideal state that patients expect to achieve, and the graduated question method is used to set up specific and feasible goals. c. Exploring patients’ advantages and providing individualized interventions: According to the patient's condition, those of his or her, family and external conditions, the patients' previous successful experience and personal advantages are explored in interviews. Targeted interventions are implemented according to his or her problems (such as targeted guidance for blood pressure control, etc.), and positive feedback is given; d. Evaluating progress: Patients’ behavior to achieve goals is explored. Patients are helped to experience a sense of achievement, and the scale scoring method is used to evaluate progress.

After the completion of each intervention, the medical staff will record the patient's care diary on the cloud platform. After two months of continuous intervention, patients' individualized problems will be basically saturated. Then we may build a patient-specific problem-solving self-management library based on the care diary of the staff and will send each patient's solution library in the form of a manual after the last intervention. In this way, even when the project is completed and the wearable device is withdrawn, the process can potentially help patients develop good self-management habits, laying a foundation for maintaining high-quality self-management in the future.

The content of the intervention will be determined according to the patient's individual problem, including but not limited to the following:

| Name | Objective | Content |
| --- | --- | --- |
| Stroke-related underlying diseases and nursing | To reduce the impact of basic diseases on stroke and promote disease recovery | Hypertension, diabetes, hyperlipidemia, heart disease, and other related conditions: identification, measurement, impact on stroke, how to use medications, common side effects, causes of poor treatment, medication errors, proper diet, exercise, complications |
| Influence and correction of behavioral factors | To reduce the impact of behavioral risk factors on disease | The harm of smoking, excessive drinking, excessive diet, fatigue and other bad behaviors to cerebrovascular diseases;  The impact of effective exercise on improving cerebrovascular diseases;  The influence of compliance behavior on the disease |
| Coping with abnormal psychological status | To identify abnormal psychological status and dealing with it positively | Identification of abnormal psychological problems such as anxiety and depression;  Guidance for psychological problems |
| Sleep | To identify sleep problems and deal with them effectively | Identification of sleep problems;  How to promote effective sleep |
| Rehabilitation | To improve impaired function and quality of life | The principle of rehabilitation;  Factors affecting rehabilitation;  Dysphagia, dressing and undressing training, transfer training (crutches, wheelchairs, going up and down stairs, eating, defecation and urination), bed functional exercise, sitting functional exercise, etc. |
